# 

**Published:** 1894-05-19

**Authors:** 


**Notices of Births, Deaths, and Marriages** are inserted in this column at a charge of 2s. 6d. for an announcement not exceeding four lines.

DEATH.

In Memoriam.

In dear memory of my loving little adopted son Jackie, who was called away on Trinity Sunday, 1891. For ten years he was my comfort and joy. A child whom every eye that looked on loved.—S. B., U.S.A.

**Notice to Advertisers and Subscribers.**

All Advertisements, Orders for Copies of The Hospital, and Business Communications should be addressed to **The Publisher, NOT TO THE EDITOR**, The Hospital, 428, Strand, W.C.

The Cost of Subscription, post free, is as follows:—Per week, 2½d.; per quarter, 2s. 8d.; per half-year, 5s. 3d.; per annum, 10s. 6d. Foreign:—Per Annum, 12s. 8d.; payable, in all cases, in advance.

NOTICE TO CORRESPONDENTS.

All MS., Letters, Books for Review, and other matters intended for the Editor should be addressed The Editor, The Lodge, Porchester Square, London, W.

The Editor cannot undertake to return rejected MS., even when accompanied by stamped directed envelope.

**"Burdett's Hospital Annual," 1894.**

Fifth year of publication. 900 pp. Boards, gilt lettering. A most complete guide to British, Colonial, Indian, and American Hospitals, Dispensaries, Nursing and Convalescent Institutions, and Asylums. Price 5s. Published by The Scientific Press (Limited), 428, Strand, W.C.

**NOTICE.—The Index for Vol. XIV. (ending Sept. 30th, 1893) is on sale at the Office of this Journal, price 2½d., post free.**

### SCRAPS AND GLEANINGS.

The Duke of York presided at the festival dinner of the Hospital for Sick Children on Saturday, May 5th.

The Earl of Derby has presented £100 to the funds of the Cottage Hospital now in course of erection at Fleetwood.

The Marquis Camden has consented to become president of the North-West London Hospital. Kentish Town Road.

An expenditure of £9,750 has been authorized by the Local Government Board in connection with the purchase of an ambulance steamboat.

The Virginia Legislature has a bill pending to cause the appointment of a proper number of women physicians to each hospital for the insane.

The Duchess of Albany has sent to the North-Eastern Hospital for Children, Hackney Road, a consignment of toys which belonged to her two children.

A large bazaar was recently held at Kershaw, the proceeds of which were devoted to the purpose of endowing a cot in the children's hospital of that locality.

Further subscriptions continue to be urged in favour of the funds of the North-Eastern Hospital for Children, a debt of nearly £4,500 existing on the accounts.

The subscriptions in pence from members towards the Provident Dispensary at Bexley have gone on increasing by £20 a year till it has reached £274 last year.

A donation of £25 has been received from the Clothworkers' Company for the funds of the Paddington Green Children's Hospital, which is now being rebuilt and enlarged.

The widow of the late Professor D. Hays Agnew has given 25,000 dollars to the University of Pennsylvania. This money will be used in erecting an extension to the hospital.

A public indignation meeting, called by posters and handbills, has been held in the Mechanics' Hall, Delph, to protest against the erection of a small-pox hospital at Waters, near Delph.

A new smallpox hospital is in contemplation by the Metropolitan Asylums Board. It will be erected in the marshes on the lower Thames, the Hospital Ship and the Darenth Camp being too small.

Three additional acres of ground have been purchased by the local authorities of Leith for the infectious diseases hospitai to be erected at East Pilton, the area of which is now extended to nine acres.

Over the working of the year's expenses of the North Riding Infirmary there is a balance in favour of the revenue to the extent of £14, reducing the adverse balance at the end of the previous year to £143.

To forward the prospects of the proposed cottage hospital at Market Harborough an entertainment was organised at the Town Hall. The funds of the proposed scheme so far amount only to about £30.

The sum of nearly £6,000 was contributed towards the funds of the Middlesex Hospital on the occasion of the Prince of Wales' speech at the festival dinner which recently took place at the Metropole Hotel.

The annual meeting in connection with the Edinburgh Dispensary for Skin Diseases was held lately. Hitherto the dispensary has been supported by private subscriptions, but now an appeal is to be made to the public.

It is proposed that the Stockton and Thornaby District Nursing Association should, with the Hartlepool Hospital Governors, each contribute a grant to cover the expenses of the proposed convalescent home for the district.

The site for the proposed new hospital at Seacombe has been given by Mr. John McInnes, near the Central Park. There are already £2,000 in hand towards the erection of the building, but a further £6,000 is stated to be required.

The Suffolk Convalescent Home, Ipswich, has received a legacy of £102 from the executors of the late Mr. W. B. Long, of Hart's Hall, and will soon receive a further legacy of £500 from the late Miss E. Lathbury, of Bury St. Edmunds.

A deficiency of £94 exists on the annual financial accounts of St. Paul's Eye and Ear Hospital, Liverpool. Adding this to the already existing debt, a total deficiency is thus left of £417 on the working expenses of the institution.

The building of the cottage hospital at Accrington is absorbing much attention on the part of the promoters of the scheme. The establishment, fully equipped, will cost about £3,000, and the maintenance is set down at £50 a bed or £600 a year.

The annual meeting of the Abingdon Cottage Hospital was recently held. A very satisfactory balance-sheet was presented; the expenditure has been well within the income, £495 having been registered for the expenses of the year's working.

The cost of the "Fountain" Temporary Hospital at Lower Tooting has been found to amount to the startling sum of about £117,000, representing a sum per head which is twice as much as that of the North Eastern Temporary Hospital at Tottenham.

A series of entertainments have been held in the New Town Hall Bournemouth, in aid of the Royal Victoria Hospital. The endowment fund of this institution only realises £88 per annum, and the annual subscription list does not exceed £1,000, therefore £1,200 has to be raised by special means.

The annual meeting of the Leicester and Leicestershire Provident Dispensary took place recently, when renewed testimony was given to the undiminished success and popularity of the Institution. There are now 38,142 members herein incorporated. The monthly payments of members amount to £5,189.

From the annual report of the Kenilworth Convalescent Home presented last week at the meeting of subscribers, it appears that 359 patients have been admitted during the year into this home. A balance of £118 is being carried over on the working expenses of the twelve months under review.

A protest against the erection of a fever hospital at Chingford, the railway terminus to Epping Forest, has been drawn up and signed by London representatives in Parliament. Epping Forest has long been a favourite recreation ground, and the choice of this spot for the erection of an infectious diseases hospital appears highly unsuitable.

### Medical Vacancies and Appointments.

### VACANCIES.

### Resident Medical Officers.

Blackburn and East Lancashire Infirmary.—Junior House Surgeon; doubly qualified. Salary £50 per annum, with board, washing, and lodging. Applications to Nathan A. Smith, Secretary, 15, Richmond Terrace, Blackburn, by May 24th.

Durham County Hospital.—House Surgeon. Salary £100 per annum, with board and lodging. Appointment for two years. Applications to V. K. Cooper, Honorary Secretary, 16, South Bailey, Durham, by June 1st.

Hospital for Sick Children, Great Ormond Street, Bloomsbury, W.C.—House Physician and House Surgeon, unmarried. Appointments for six months. Salary, in each case, £20, with board and residence in the hospital. Applications to the Secretary by May 25th.

Lancaster Infirmary and Dispensary.—House Surgeon; unmarried, doubly qualified. Salary £80 per annum, with residence, board; attendance, and washing. Applications, on forms to be obtained of the Secretary, to the Secretary by May 23rd.

Nottingham Borough Asylum.—Second Assistant Medical Officer, unmarried. Salary £100 per annum, with board, apartments, and washing. Applications to the Medical Superintendent by May 21st.

Royal Cornwall Infirmary, Truro.—House Surgeon; doubly qualified. Salary £120 per annum, increasing £10 yearly to £150, with furnished apartments, fire, light, and attendance. Applications to the Secretary by May 21st.

Stamford, Rutland, and General Infirmary.—House Surgeon; unmarried; doubly qualified. Salary £100 per annum, with board, lodging, and washing. Applications to the Chairman of the Special Committee by May 25th.

Vestry of St. Margaret and St. John, Westminster.—Medical Officer; not less than 25, or more than 45, years of age. Salary £250 per annum. Applications, marked on the envelope "Medical Officer," to be delivered at the Town Hall, Westminster, S.W., by May 21st.

### Non-Resident Medical Officers.

London County Council.—Coroner for the North-Eastern District of London; not under 35 nor more than 50 years of age. Salary £1,150 per annum. Applications, marked outside "Coroner for N.E. District," to the Clerk of the Council, Spring Gardens, S.W., by May 21st.

National Hospital for Diseases of the Heart and Paralysis, 32, Soho Square, W.—Physician. Applications to the Secretary.

St. Luke's Hospital.—Two Clinical Assistants. Appointment for six months. Board and residence provided. Applications to Percy De Bathe, M.A., Secretary, by May 22nd.

University College, Liverpool.—George Holt Chair of Pathology and Derby Chair of Anatomy. Endowment, £375 per annum each, with share of fees. Applications to the Registrar by June 2nd.

Westminster Hospital, Broad Sanctuary, S.W.—Surgical Registrar. Must be F. or M.R.C.S.Eng. Appointment for twelve months. Salary £40 per annum. Applications to Sidney M. Quennell, Secretary, by May 22nd.

### The Hospital Advertiser.

### MEDICAL APPOINTMENTS.

**STAMFORD, RUTLAND, AND GENERAL INFIRMARY.**

### THE SCIENTIFIC PRESS LIST.

**MEDICAL HISTORY FROM THE EARLIEST TIMES.** By E. T. WITHINGTON, M.A., M.B., Oxon. Demy 8vo., about 400 pp. [*Immediately.*

**HOSPITALS, DISPENSARIES, AND NURSING.** 500 pp. 60 illustrations. Price 21s. net.

**THE ORGANIZATION OF CHARITIES.** Cloth, gilt, 400 pp. Price 6s. net.

**THE MOTHER'S HELP AND GUIDE TO THE DOMESTIC MANAGEMENT** OF CHILDREN. By P. MURRAY BRAIDWOOD, M.D. Price 2s. 6d.

**HELPS IN SICKNESS AND TO HEALTH.** By HENRY C. BURDETT. Illustrated, Crown 8vo., 400 pp., nearly ready, price 5s.

**A PRACTICAL HANDBOOK OF MIDWIFERY.** By FRANCIS W. N. HAULTAIN, M.D. 18mo., profusely illustrated with original cuts, tables, &c.; about 260 pp., handsomely bound in leather. Price 6s.

**COTTAGE HOSPITALS: General, Fever, and Convalescent.** By HENRY C. BURDETT. (Third Edition in the Press.)

**SPINAL CURVATURE AND AWKWARD DEPORTMENT: their Causes and** Prevention in Children. Dr. GEORGE MULLER, Professor of Medicine and Orthopædics, Berlin. English Edition, edited and adapted by Richard Greene, F.R.C.P. Crown 8vo,, cloth gilt, illus., 2s. 6d. [*In the Press.*

**MYXŒDEMA; and the Effect of Climate on the Disease.** By A. MARIUS WILSON, M.D. Cloth gilt. Price 2s.

**HOSPITALS AND ASYLUMS OF THE WORLD:** Their Origin History, Construction, Administration, Management, and Legislation; with Plans of the Chief Hospitals, Asylums, Convalescent Institutions, Special and Infectious Hospitals, Nurses' Homes, and Medical School Buildings, in addition to those of all the Hospitals of London in the Jubilee Year of Queen Victoria's Reign, accurately drawn to a Uniform Scale. By HENRY C. BURDETT. In Four Volumes; Crown quarto, handsome cloth gilt, bevelled edges, profusely illustrated with plans and designs. Together with a portfolio containing some hundreds of plans. Price £8 8s. complete.

**OPHTHALMIC NURSING.** An important work on a new subject, profusely Illustrated. By SYDNEY STEPHENSON, M.B., F.R.C.S.E. With list of instruments required, glossary, &c. Crown 8vo., 200 pp. Price 3s. 6d.

**SURGICAL WARD-WORK AND NURSING.** A Practical Manual of Clinical Instruction for Nurses and Students. By ALEXANDER MILES, M.D. (Edin.), C.M., F.R.C.S.E. Over one hundred illustrations. Demy 8vo , 200 pp. Price 3s. 6d.

**HOSPITAL SISTERS AND THEIR DUTIES.** By Eva C. E. Luckes. 2nd Edition. 2s. 6d.

**THE THEORY AND PRACTICE OF NURSING.** By PERCY G. LEWIS, M.D. 6th Edition. 3s. 6d.

**MENTAL NURSING.** 2nd Edition. By WILLIAM HARDING, M.D. 2s. 6d.

**OUTLINES OF INSANITY.** By FRANCIS H. WALMSLEY, M.D. Popular Edition. 1s. 6d.

**ART OF FEEDING THE INVALID.** By a MEDICAL PRACTITIONER and a LADY PROFESSOR OF COOKERY. 3s. 6d.

**ART OF MASSAGE.** By A. CREIGHTON HALE. 70 illustrations. 6s.

**THE NURSE'S DICTIONARY OF MEDICAL TERMS AND NURSING** TREATMENT. By HONNOR MORTEN. 15th thousand. Cloth, 2s.; leather, 2s. 6d.

**BURDETT'S HOSPITAL ANNUAL, and Year-book of Philanthropy, 1894.** By HENRY C. BURDETT. 5s.

**ACCOUNTS, AUDIT, AND TENDERS, for *Hospitals and Institutions*,** The Uniform System of. By HENRY C. BURDETT. 6s.

**ACCOUNT BOOKS FOR INSTITUTIONS.** Series No. 1: Complete set of Eight Books, price £10. Series No. 2: Set of Four Books (for small Institutions), price £4. Full catalogue and particulars post free.

**SUFFERING LONDON: A Book about Hospitals and their Needs.** By A. EGMONT HAKE, with an Introduction by Walter Besant. Cloth, 250 pp. Price 3s. 6d.

**LECTURES ON GENITO-URINARY DISEASES.** By J. C. OGILVIE WILL, *M.D., C.M., F.R.S.E. Demy 8vo., about 150 pp., profusely illustrated with coloured and other plates and drawings.* Price 6s. net.

**HOW TO BECOME A NURSE.** By HONNOR MORTEN. Illustrated. Price 2s. 6d.

***MINISTERING WOMEN: The Story of the Royal National Pension Fund for*** Nurses. By GEORGE W. POTTER, M.D. 2s. 6d.

**"THE HOSPITAL" MONTHLY PART FOR MAY.** Containing about 200 pp. Illustrated. Paper covers. Price 6d.; or by post, 8½d.

London: THE SCIENTIFIC PRESS (Limited), 428, Strand, W.C.

